# FOXM1 regulates proliferation, senescence and oxidative stress in keratinocytes and cancer cells

**DOI:** 10.18632/aging.100988

**Published:** 2016-07-03

**Authors:** Artem Smirnov, Emanuele Panatta, AnnaMaria Lena, Daniele Castiglia, Nicola Di Daniele, Gerry Melino, Eleonora Candi

**Affiliations:** ^1^ University of Rome “Tor Vergata,” Department of Experimental Medicine and Surgery, 00133, Rome, Italy; ^2^ Istituto Dermopatico dell'Immacolata (IDI-IRCCS), 00166, Rome, Italy; ^3^ University of “Tor Vergata”, Department of Systems Medicine, 00133, Rome, Italy

**Keywords:** FOXM1, head and neck cancer, skin, p63, oxidative stress, senescence

## Abstract

Several transcription factors, including the master regulator of the epidermis, p63, are involved in controlling human keratinocyte proliferation and differentiation. Here, we report that in normal keratinocytes, the expression of FOXM1, a member of the Forkhead superfamily of transcription factors, is controlled by p63. We observe that, together with p63, FOXM1 strongly contributes to the maintenance of high proliferative potential in keratinocytes, whereas its expression decreases during differentiation, as well as during replicative-induced senescence. Depletion of FOXM1 is sufficient to induce keratinocyte senescence, paralleled by an increased ROS production and an inhibition of ROS-scavenger genes (SOD2, CAT, GPX2, PRDX). Interestingly, FOXM1 expression is strongly reduced in keratinocytes isolated from old human subjects compared with young subjects. FOXM1 depletion sensitizes both normal keratinocytes and squamous carcinoma cells to apoptosis and ROS-induced apoptosis. Together, these data identify FOXM1 as a key regulator of ROS in normal dividing epithelial cells and suggest that squamous carcinoma cells may also use FOXM1 to control oxidative stress to escape premature senescence and apoptosis.

## INTRODUCTION

Human keratinocytes undergo terminal differentiation by migrating from the inner basal layer to the outer cornified layer to form the epidermis. Several transcription factors, including the master regulator of the epidermis, p63 [1–3] but also c-MYC, ZNF750, and KLF4 [4, 5], are involved in regulating this complex cellular program. p63, in particular the p63 amino-deleted isoform (ΔNp63), is highly expressed in epithelial progenitor cells, where it is indispensable to maintain the proliferative potential of different organs, including the epidermis, thymus, and prostate, as well as in glandular structures [6–10]. In fact, both p63KO and ΔNp63KO mice display depleted epithelial stem cell reserves and ectodermal-derived cell failure, leading to severe limb, craniofacial, skin and skin appendage developmental defects [7, 8, 11, 12].

In proliferating keratinocytes, ΔNp63 is also involved in different cellular programs through the activation of specific sub-sets of target genes, including genes relevant for epidermal formation, cell adhesion and anti-oxidant genes [7, 8, 13–26]. Among the latter genes, p63 controls the expression of glutathione peroxidase (GPX, [27]), REgulated in development and DNA Damage Response 1 (REDD1, [28]), cytoglobin (CYB, [26]), hexokinaseII (HK, [25]) and glutaminase-2 (GLS2, [29]).

Experiments (RT-qPCR-based microarray and RNA-seq, [25, 26]) previously performed in our laboratory indicated that the expression of the transcription factor FOXM1 is strongly reduced in p63-depleted keratinocytes. FOXM1 is a member of the Forkhead domain protein family, which includes almost 100 different transcriptional factors involved in a broad range of processes [30–32], including stem cell expansion and renewal [33]. In particular, FOXM1 controls a network of genes necessary for G_2_-M phase transition and cell division [34]. FOXM1 is expressed during embryonic development, particularly in proliferative epithelia and mesenchymal cells [35]. In addition, FOXM1 has been shown to play an important role in oxidative stress regulation. Knocking down FOXM1 in fibroblast leads to increased intracellular levels of ROS. Catalase and superoxide dismutase are also known to be direct transcriptional targets of FOXM1 [36]. FOXM1 level is the most common differentially expressed gene in the majority of cancers, including oral, esophageal, lung, breast, kidney, and bladder cancer, as compared to normal tissues, suggesting a possible role in cancer initiation [37, 38].

To date, studies of FOXM1 have been performed only in the context of cancer cell lines and/or immortalized cell lines in which the aberrant genetic background interferes with FOXM1 function. Herein, we sought to investigate the link between p63 and FOXM1 in normal human epidermal keratinocytes (NHEKs) by examining the effect of p63 deletion using specific siRNAs. We demonstrated that ΔNp63 indirectly regulates FOXM1 expression. Both transcription factors are important to maintain the proliferative potential of keratinocytes and to control the cellular redox state. FOXM1 depletion sensitizes normal keratinocytes and head and neck squamous carcinoma cells to apoptosis. Interestingly, FOXM1 knock-down induces replicative senescence, and skin biopsies of old subjects present reduced FOXM1 mRNA and protein levels compared with young subjects. Altogether, the data presented indicate that ΔNp63 and FOXM1 are important in maintaining and protecting skin progenitor cells to allow proper skin homeostasis and to counteract cellular senescence.

## RESULTS

### ΔNp63 indirectly regulates FOXM1 expression

**Figure 1:**
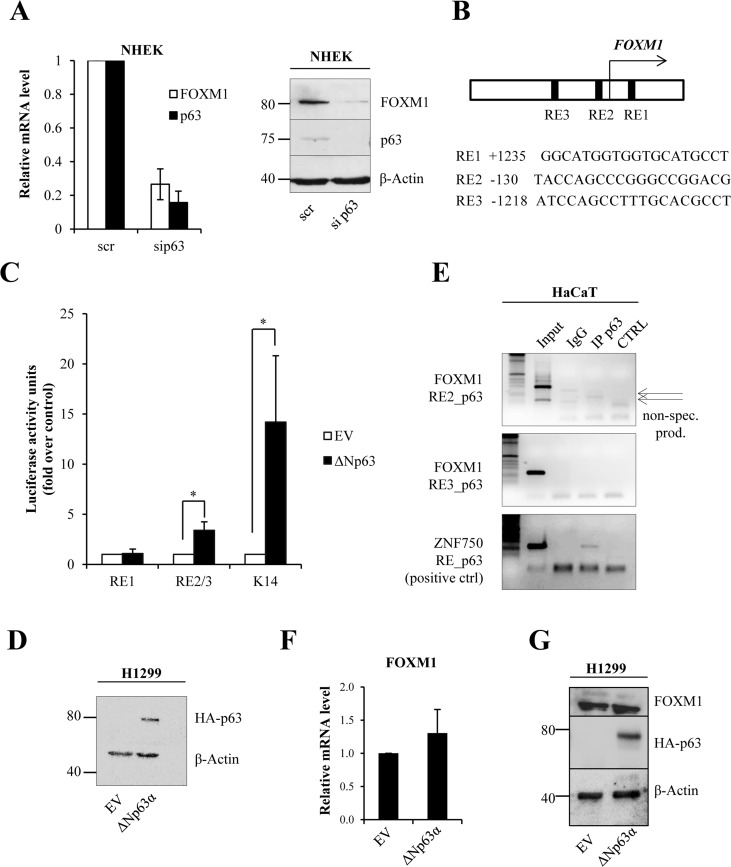
ΔNp63 indirectly regulates FOXM1 expression (**A**) NHEKs were silenced for p63, and the relative mRNA (48 h after transfection) and protein (96 h after transfection) levels of FOXM1 and p63 were determined. Values reported are the average ± SD of three independent experiments. (**B**) Scheme of the FOXM1 promoter showing the identified putative p63 response elements (REs). (**C**) Luciferase activity assay in H1299 cells transfected with pGL3 vectors containing putative response elements from the FOXM1 promoter. Values reported are the average ± SD of three independent experiments. *p-value <0.05 by Student's *t*-test. (**D**) Western blot analysis of the lysates that were used for luciferase assays. (**E**) Chromatin immunoprecipitation was performed in HaCaT cells with anti-p63 antibody (IP p63) or negative control immunoglobulin G (IgG). PCR was carried out with specific primers for putative p63 response elements in the FOXM1 promoter (RE2 and RE3). The ZNF750 promoter was used as a positive control for immunoprecipitation. The arrows indicate non-specific products. (**F**) H1299 cells were transfected with pcDNA vector expressing ΔNp63α–HA. After 24 h, FOXM1 expression levels were analyzed by qPCR. (**G**) Western blot showing ΔNp63α–HA expression and FOXM1 level.

Our previous RT-qPCR-based microarray and RNA-seq experiments [25, 26] revealed that FOXM1 expression is strongly down-regulated in p63-depleted keratino-cytes. We confirmed the results of the array in human keratinocytes (Fig 1A) demonstrating that siRNA-mediated knock-down of p63 in keratinocytes leads to decreased FOXM1 mRNA and protein levels. Next, we performed bioinformatic analyses to identify putative p63 response elements in the *FOXM1* promoter. We identified one putative response element downstream of the transcription start site (TSS) and two that were upstream of the TSS (Fig 1B). We constructed vectors containing the sequences of these putative p63 response elements based on pGL3-vectors. Then, we carried out luciferase activity assays in H1299 cells overexpressing ΔNp63. As a positive control of ΔNp63 transcriptional activity, we used a pGL3 vector containing the keratin14 (K14) promoter sequence [9, 20]. We did not observe any strong activation of luciferase expression with the FOXM1 promoter as compared to p63-target keratin14 (approximately 3-fold for RE2/3 versus approximately 15-fold for the K14 promoter, Fig 3C-D). A mild activation of luciferase expression may indicate that some other transcriptional factors specific for keratinocytes are needed for p63-dipendent regulation of FOXM1 expression. We also confirmed this result performing chromatin immunoprecipitation and analyzing ChIP-seq data [21]: no p63 binding was detected on the indicated REs of the FOXM1 promoter (Fig 1E and S1). As a positive control, we used primers for ZNF750, which is known to be a direct target of p63 [5]. In addition, we overexpressed ΔNp63 in H1299 cells and analyzed *FOXM1* mRNA expression, but we did not observe any changes (Fig 1F-G). All these data indicate that p63 regulates FOXM1 indirectly.

### FOXM1 levels correlate with proliferation status in keratinocytes

**Figure 2:**
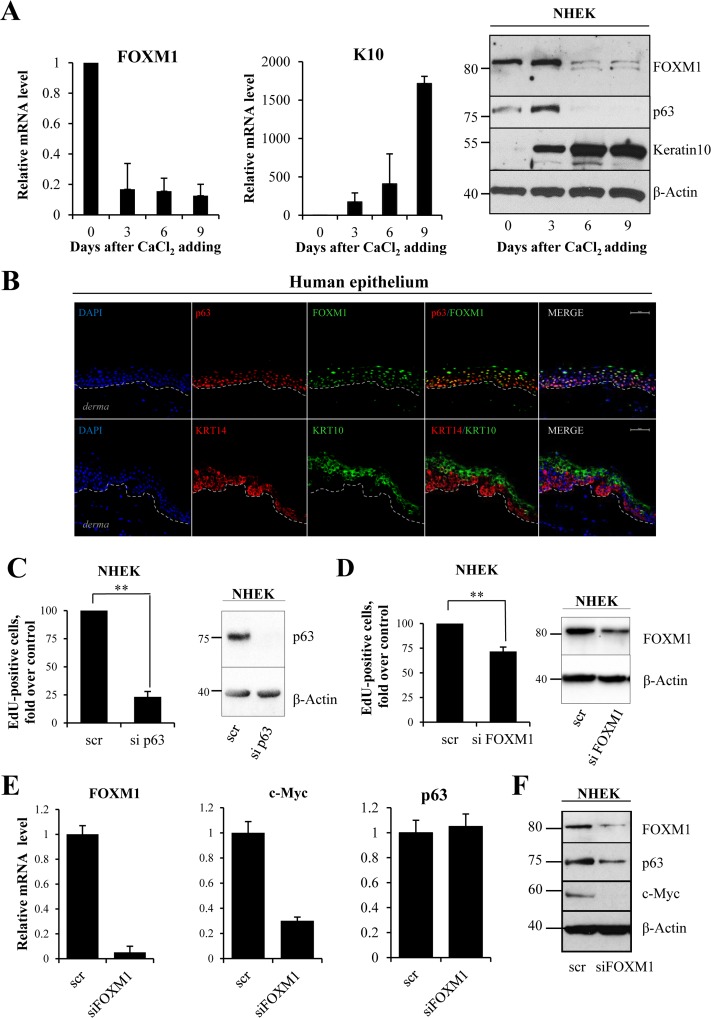
FOXM1 level correlates with proliferative status of keratinocytes (**A**) NHEKs were treated with calcium chloride to induce differentiation. FOXM1, Keratin10 and p63 levels were analyzed at 0, 3, 6 and 9 days after treatment by qRT-PCR and western blotting. Values reported are the average ± SD of four independent experiments. (**B**) Human epithelium was stained for FOXM1, p63 (lower layer marker), KRT10 (upper layers marker) and KRT14 (lower layer marker) and analyzed with confocal microscopy. DAPI was used for nucleus staining. The bar indicates 25 um. (**C**) NHEKs were silenced for p63 for 72 h, after which EdU-incorporation assays were performed. The percentage of EdU-positive cells was analyzed by FACS. (**D**) NHEKs were silenced for FOXM1 for 96 h, after which EdU-incorporation assay was performed. The percentage of EdU-positive cells was analyzed by FACS. Western blots confirm the silencing. Values reported are the average ± SD of two (for p63) and three (for FOXM1) independent experiments. **p-value <0.01 by Student's *t*-test. (**E**) Cells were silenced for FOXM1 for 96 h, after which the relative expression levels of *FOXM1, c-MYC,* and *p63* were determined by qRT-PCR. (**F**) Western blot analysis of FOXM1, p63, and c-MYC levels.

To investigate the function of FOXM1 in keratinocytes, we studied FOXM1 expression in calcium-induced differentiation. qRT-PCR and western blot analysis showed a dramatic decrease in *FOXM1* mRNA and protein levels (Fig 2A) during keratinocyte differentiation. As a positive control, we measured keratin10 (K10) expression. Interestingly, in human epidermis FOXM1 co-localizes with p63 in progenitor layer of epidermis (Fig 2B). To evaluate the role of FOXM1 as down-stream mediator of p63, we performed p63 and FOXM1 knock-down and measured the percentage of cells in S-phase. EdU-incorporation assays in NHEKs and in spontaneously immortalized (HaCaT) keratinocytes (Fig 2D and Fig S3) indicated a 25% decrease in EdU-positive cells (from 18.0±3.3% to 12.5±3.3% in NHEK) upon siFOXM1, whereas sip63 caused an 80% reduction (Fig 2C-D, Fig S2). This difference could also due in part to knock-down efficacy that may be explained by higher protein stability of FOXM1 as has been shown previously [39, 40]. As a control, we performed qRT-PCR and western blot analysis for c-Myc, whose expression is in part controlled by FOXM1 [41], confirming a link between FOXM1 and c-Myc (Fig 2E,F). Thus, we propose the presence of a positive feedback loop between FOXM1, p63 and c-Myc in controlling keratinocyte proliferation. Altogether, these data indicate that FOXM1 is necessary in normal dividing epithelial cells.

### FOXM1 regulates oxidative stress and ROS-mediated cell death in keratinocytes

**Figure 3:**
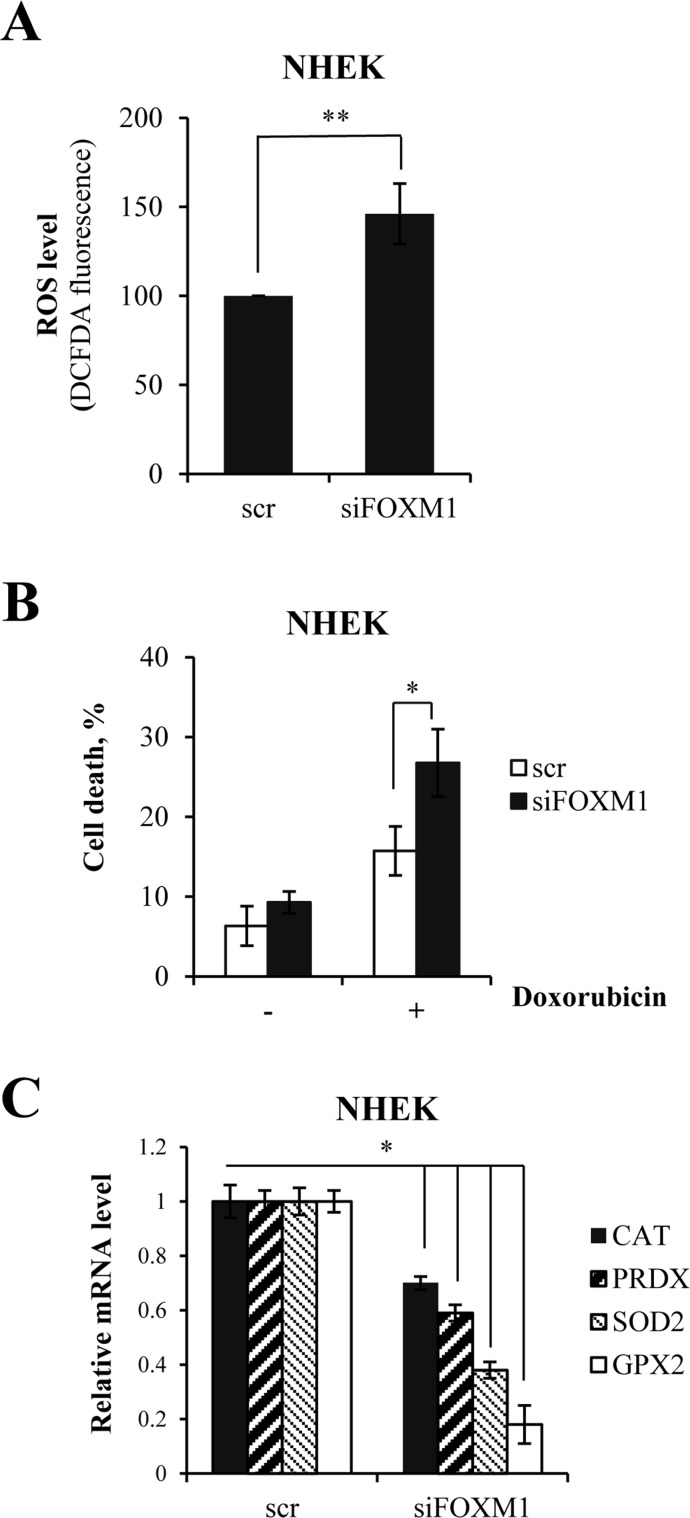
FOXM1 regulates oxidative stress and ROS-mediated cell death in keratinocytes (**A**) Cells were silenced for FOXM1 for 96 h. ROS levels were then measured by FACS. Values reported are the average ± SD of three independent experiments. **p-value <0.01 by Student's *t*-test. (**B**) Cells were silenced for FOXM1 for 96 h and treated with 1 μM doxorubicin. The percentage of sub-G1 events was measured by FACS at 24 h after treatment. Values reported are the average ± SD of three independent experiments. *p-value <0.05 by Student's *t*-test. (**C**) Cells were silenced for FOXM1 for 96 h, after which the relative expression levels of *CAT, PRDX, SOD2,* and *GPX2* were determined by qRT-PCR. Values reported are the average ± SD of two independent experiments. *p-value <0.05 by Student's *t*-test.

We predicted that FOXM1 might protect keratinocytes from oxidative stress and ROS-mediated cell death. In order to verify this prediction, we measured intracellular ROS following siRNA-mediated knock-down of FOXM1 in keratinocytes. We observed a 1.5-fold increase in ROS levels in FOXM1-knock-down cells (Fig 3A). Then, we exposed cells to oxidative stress using doxorubicin, which is known to produce reactive oxygen species. We observed a significant increase in cell death in FOXM1-depleted cells compared with control cells (Fig 3B). In parallel, qRT-PCR analysis revealed the decreased expression of genes encoding proteins involved in oxidative stress responses (e.g., *CAT, PRDX, SOD2,* and *GPX2*) (Fig 3C). Altogether, these data showed that FOXM1 participates in balancing intracellular ROS level and protects keratinocyte from oxidative stress.

### FOXM1 levels decrease during keratinocyte senescence and during skin aging

**Figure 4:**
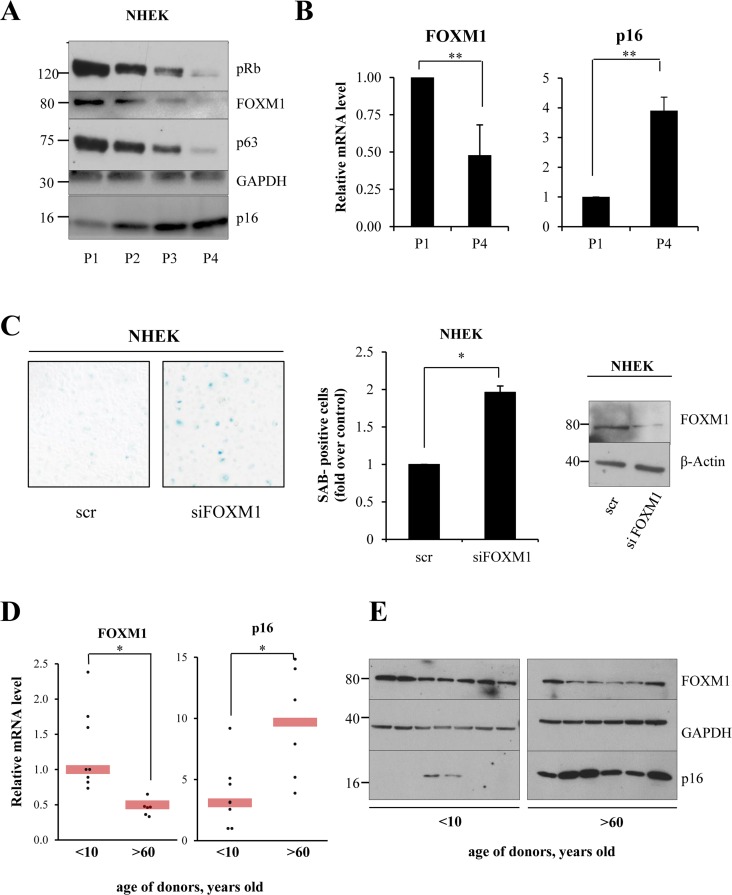
FOXM1 levels decrease during keratinocyte senescence (**A**) NHEKs were maintained for 4 passages, and FOXM1, p63, Rb, and p16 levels were analyzed for each passage by western blot. (**B**) qPCR analysis of *FOXM1* and *p16* expression levels in proliferating (P1) and senescent (P4) keratinocytes. Values reported are the average ± SD of three independent experiments. **p-value <0.01 by Student's *t*-test (**C**) NHEKs were silenced for 96 h, after which SA-β-galactosidase assays were performed. Values reported are the average ± SD of two independent experiments. *p-value <0.005 by Student's *t*-test. **(D)** qRT-PCR analysis of *FOXM1* and *p63* expression levels in primary keratinocytes obtained from biopsies of young (<10 y.o.) and aged (>60 y.o.) donor skin. *p-value <0.005 by Student's *t*-test. (**E**) Western blot analysis of FOXM1 and p16 levels in keratinocytes grown as in (**D**).

Having shown that FOXM1 has a role in protecting cells from oxidative stress, we next evaluated whether FOXM1 protects cells against senescence. We used a previously described [42] in vitro cellular model of replicative senescence, based on serial passaging of primary keratinocytes (P1, P2, P3, and P4). In this model, at P4, the cells undergo replicative-induced senescence, as determined by morphology, expression markers, and SA-beta-galactosidase activity. We analyzed the levels of FOXM1 in proliferating (P1) and senescent (P4) cells and observed a decrease in FOXM1 mRNA and protein levels (Fig 4A-B) in senescent-induced cells. Senescence was marked using p16, phosphorylated Retinoblastoma protein (pRb) and p63 (Fig 4A-B). To investigate whether FOXM1 depletion is sufficient to induce senescence in proliferating keratinocytes, we performed siRNA-mediated knock-down of FOXM1 in P1 keratinocytes and evaluated the senescence-associated-β-galactosidase activity. As indicated in Fig 4C, the results showed a two-fold increase in β-galactosidase-positive cells in knocked-down samples compared with controls. Interestingly, FOXM1 levels were also down-regulated during skin natural aging in humans. We performed analysis of keratinocytes obtained from biopsies of young (<10 years old) and aged (>60 years old) donors and observed a 50% decrease in *FOXM1* mRNA levels, which was paralleled by a decrease in protein levels (Fig 4D-E). p16 served as positive control. These results indicate that FOXM1 expression significantly decreases during senescence in keratinocytes both *in vitro* and *in vivo* and that FOXM1 activity counteracts replicative-induced senescence.

### FOXM1 regulates oxidative stress in epithelial squamous cell carcinoma

**Figure 5:**
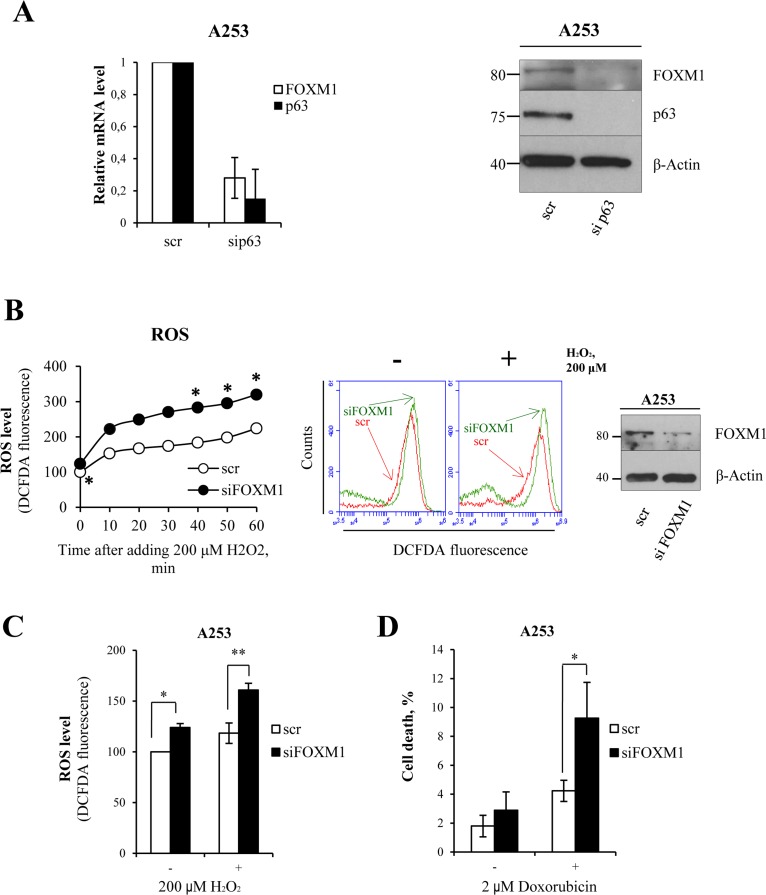
FOXM1 regulates oxidative stress in epithelial squamous cell carcinoma **(A)** A253 cells were silenced for p63, and relative mRNA (48 h after transfection) and protein (96 h after transfection) levels of FOXM1 and p63 were determined. Values reported are the average ± SD of two independent experiments. **(B)** Time-course analysis of ROS levels in A253 cells silenced for FOXM1 for 96 h and treated with 200 μM hydrogen peroxide. The ROS level was measured by FACS. Values reported are the average of two independent experiments. *p-value <0.05 by Student's *t*-test. Western blot confirming the silencing. **(C)** Cells were silenced for FOXM1 for 96 h and treated with 200 μM hydrogen peroxide. The ROS level was measured by FACS at 24 h after treatment. Values reported are the average ± SD of three independent experiments. *p-value <0.05 and **p-value <0.001 by Student's *t*-test. **(D)** Cells were silenced for FOXM1 for 96 h and treated with 2 μM doxorubicin. The percentage of sub-G1 events was measured by FACS at 24 h after treatment. Values reported are the average ± SD of three independent experiments. *p-value <0.05 by Student's *t*-test.

In order to investigate whether p63 and FOXM1 are also linked in epithelial cancer, we performed siRNA-mediated knock-down of p63 in the A253 cell line (submaxillary salivary gland epidermoid carcinoma). The results showed a significant decrease in FOXM1 at both the mRNA and protein levels (Fig 5A), indicating that p63 also indirectly controls FOXM1 expression in cancer cells. To study the physiological role of FOXM1 in this type of cancer, we performed siRNA-mediated knock-down of FOXM1. We exposed the cells to oxidative stress and measured ROS levels after exposure. Analysis of ROS levels a short time after hydrogen peroxide addition showed a significant increase in ROS levels in knocked-down samples with respect to controls (Fig 5B). We also analyzed ROS levels 24 h after treatment with hydrogen peroxide, which showed the same effect (Fig 5C). Furthermore, we observed an increase in cell death in knocked-down samples with respect to the control at 24 h after treatment with doxorubicin (2-fold, Fig 5D). These results indicated that the p63-FOXM1 axis also has a protective role in cancer cells. Because different cancer types express high levels of FOXM1, FOXM1 anti-oxidant activity could be a mechanism through which cancer cells escape premature senescence and apoptosis.

## DISCUSSION

**Figure 6:**
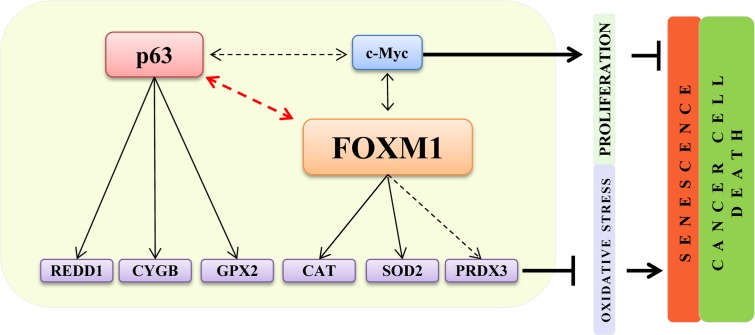
A FOXM1- and p63-dependent positive feedback loop FOXM1 and p63, due to the positive feedback loop, are both involved in the regulation of oxidative stress responses and proliferation in normal epithelial cells. FOXM1 acts as a sensor and regulator of oxidative stress to protect normal and tumor proliferating cells.

Senescence is characterized by a progressive decline of cellular and body homeostasis [43–45]. When premature senescence acts cellular systems are not able to adequately respond to stress stimuli resulting in age-related diseases [46–50]. FOXM1 is an important transcription factor that controls genes directly involved in cell cycle control and in the successful execution of the mitotic program, as well as in the maintenance of chromosome stability [51]. Studies have demonstrated that FOXM1 is required for the expansion of epithelial progenitor cells and that UV-light and environmental factors, such us nicotine, can directly activate the FOXM1 transcriptional network in keratinocytes [34], suggesting that FOXM1 could be an oncogenic hit. In fact, FOXM1 is overexpressed in various human malignancies, including prostate, breast, lung, ovary, colon, pancreas, stomach, bladder, liver and kidney cancer [52, 53], and its oncogenic activity is downstream of Ras [54] and cyclin D1 [38], which are often implicated in epithelial tumors. In addition, FOXM1 has been shown to counteract oxidative stress-induced premature senescence by stimulating Bmi [55]. Additional studies using immortalized MEFs demonstrated that FOXM1 expression is induced by oncogenic stresses requiring ROS and that up-regulated FOXM1 engages a negative feedback loop to counteract ROS increases and protect dividing and cancer cells from oxidative stress [36]. Here, we investigated the role of FOXM1 in normal human primary keratinocytes. Important role in primary epithelial cells. We found that under normal conditions, ΔNp63 indirectly controls FOXM1 expression and that both factors are important to maintain the high proliferation rate of epithelial progenitor cells. FOXM1 also protects keratinocytes from apoptosis-induced oxidative stress by directly inducing the expression of anti-oxidant genes. Interestingly, FOXM1-depletion is sufficient to induce cellular senescence in proliferating epithelial cells, partially because of increased intracellular ROS (Fig 6). These *in vitro* results nicely paralleled the FOXM1 down-regulation (at the mRNA and protein level) measured in keratinocytes isolated from young and aged human donors biopsies. The latter results indicate that FOXM1 plays an important role, similar to that of p63, in maintaining normal tissue homeostasis and to avoid premature cellular senescence and, possibly, organismal aging (Fig 6). Interestingly, FOXM1 also regulates c-myc expression which has been shown to prevent cellular senescence [56–58] suggesting that both p63 and c-myc contribute to counteract cellular senescence in FOXM1-dipendent fashion. Although FOXM1 overexpression in various human malignancies has been very well described [52, 53], no information is available regarding the mechanisms that controls FOXM1 over-expression. Our data indicated that the p63-FOXM1 axis is also present in submaxillary salivary gland epidermoid carcinoma (A253) cells, suggesting that ΔNp63 amplification could, at least in part, be responsible for FOXM1 over-expression in epithelial cancers. Finally, our results also indicated that squamous carcinoma cells uses FOXM1 to control oxidative stress to escape premature senescence and apoptosis.

## METHODS

### Cell culture and transfection

Neonatal normal human epidermal keratinocytes (NHEKs, Life Technologies) were cultured in EpiLife medium with human keratinocyte growth supplements added (Life Technologies). Cells were induced to differentiate by adding 1.2 mM CaCl_2_ to the culture medium. For FOXM1 and p63 siRNA-mediated knockdown, NHEK and A253 cells were transfected with specific siRNAs. The following siRNAs were used: siFOXM1_5 GTGGTCCGTAAATAGTATA (FlexiTube, Qiagen), siFOXM1_6 AACATCAGAGGAGGAACCTAA (FlexiTube, Qiagen), siFOXM1_7 TGGGATCAAGAT TATTAACCA (FlexiTube, Qiagen), siFOXM1_8 GACATTGGACCAGGTGTTTAA (FlexiTube, Qiagen), sip63 reagents were from Dharmacon (On Target Plus Smart Pool hTP63, Dharmacon). The Negative Control siRNA (Qiagen, AATTCTCCGAACGTGTCACGT) was used as a silencing control. All transfections were performed using the Lipofectamine RNAiMAX transfection reagent (Invitrogen) according to manufacturer's protocols. Human Non-Small-Cell Lung Carcinoma (H1299) cells were grown in DMEM-F12, HaCaT cells were grown in DMEM, and A253 cells were grown in McCoy's medium. Each medium contained 10% FBS, 100 U penicillin, 100 μg/mL streptomycin (GIBCO, Invitrogen).

### Western blotting

Cells were lysed with SDS lysis buffer (100 mM Tris, pH 8.8, 1% SDS, 5 mM EDTA, 20 mM DTT, and 2 mM AESBF). Total cell extracts were resolved on an SDS polyacrylamide gel using the Mini-PROTEAN Tetra cell System (Bio-Rad) and blotted onto a Hybond P PVDF membrane (GE Healthcare) using the Mini Gel Tank transfer system (Life Technologies). After being blocked with PBST 5% non-fat dry milk (Bio-Rad), membranes were incubated over night with primary antibodies at +4°C, washed and hybridized for 1 h at room temperature using the appropriate horseradish peroxidase-conjugated secondary antibody (rabbit and mouse, Bio-Rad, Hercules, California, USA). Detection was performed with the ECL chemiluminescence kit (Perkin Elmer, Waltham, Massachusetts, USA). The following antibodies were used: anti-FOXM1 (Ab184637, Abcam; dilution 1:300), anti-p63 (Ab735, Abcam; dilution 1:300), anti–β-actin (Sigma; dilution 1:30000), anti-p16 (SC-56330 (JC8), Santa Cruz Biotechnology; dilution 1:1000), anti-Rb (BD554136, BD Biosciences; dilution 1:500), anti-Keratin10 (PRB-159P, Covance; dilution 1:1000), anti-HA (16612, Covance; dilution 1:1000), anti-cMyc (SC-40 (9E10), Santa Cruz Biotechnology; dilution 1:200).

### RNA extraction and real-time PCR analysis

Total RNA was isolated using the RNeasy Mini Kit (Qiagen) following the manufacturer's protocol. Total RNA was quantified using a NanoDrop spectrophotometer (Thermo Scientific) and used for cDNA synthesis using the GoScript Reverse Transcription System (Promega); qPCR was performed with the GoTaq Real-Time PCR System (Promega), and TBP was used as a housekeeping gene for normalization (Applied Biosystems). The expression of each gene was defined from the threshold cycle (Ct), and relative expression levels were calculated by using the 2^−ΔΔCt^ method. The following primers were used:

hFOXM1-FOR 5′-TGGGGAGGAAATGCCACACTTAG-3′;
hFOXM1-REV 5′–TAGGACTTCTTGGGTCTTGGGGTG-3′;
hTBP-FOR 5′–TCAAACCCAGAATTGTTGTCC-3′; hTBP-REV 5′–CCTGAATCCCTTTAGAATAGG-3′
hKer10-FOR 5′–AGGAGGAGTGTCATCCCTAAG-3′;
hKer10-REV 5′–AAGCTGCCTCCATAACTCCC-3′; hΔNp63-FOR 5′–GAAGAAAGGACAGCAGCATTG-3′;
hΔNp63-REV 5′–GGGACTGGTGGACGAGGAG-3′;
hp16-FOR 5′–GCCGATCCAGGTCATGATGGAT-3′;
hp16-REV 5′–AGCACCACCAGCGTGTCCAG-3′; hcMyc-FOR 5′–TTCGGGTAGTGGAAAACCAGC-3′;
hcMyc-REV 5′–CCTCCTCGTCGCAGTAGAAAT-3′;
hCat-FOR 5′–TGGGGAGGAAATGCCACACTTAG-3′;
hCat-REV 5′–TAGGACTTCTTGGGTCTTGGGGTG-3′;
hPRDX-FOR 5′–TCAAACCCAGAATTGTTGTCC-3′;
hPRDX-REV 5′–CCTGAATCCCTTTAGAATAGG-3′;
hSOD2-FOR 5′–GAAGAAAGGACAGCAGCATTG-3′;
hSOD2-REV 5–GGGACTGGTGGACGAGGAG-3′;
hGPX2-FOR 5′–GCCGATCCAGGTCATGATGAT-3′;
hGPX2-REV 5′-AGCACCACCAGCGTGTCCAG-3′.

### Chromatin immunoprecipitation assay

HaCaT cells were used for ChIP assay. Cells were collected, fixed in 1% formaldehyde, and subjected to sonication for DNA shearing. The chromatin immunoprecipitation was performed with an anti-p63 antibody (H129, Santa Cruz Biotechnology) or unspecific immunoglobulin G (IgG) (Invitrogen) using a ChIP assay Kit (Invitrogen). For the amplification of the promoter region containing potential p63 response elements, the following primers were used: FOXM1_RE2-FOR 5′-AAACTCTCCCTCGGCTCGC-3′; FOXM1_RE2-REV 5′- GAAGGCTGTGCGGTCTGCC-3′; FOXM1_RE3-FOR 5′-ACAAAACTTCTCGGTATGGCTAAG-3′; FOXM1_RE3-REV 5′-GAAAGGCTTTTGTAATGAGAGCTTG-3′; ZNF750-FOR 5′–GGAGGGAGCTTATCCCAGAG-3′; ZNF750-REV 5′–CCTCCGATTAAGCAAGCAAG-3′.

### Luciferase assay and constructs

Two regions of the FOXM1 promoter (RE1, −1500 to 0, and RE2/3, +900 to +1600) were amplified from human genomic DNA by PCR and subcloned into the pGL3-Promoter (for RE1) or pGL3-Basic (for RE2/3) reporter vectors (Promega), which had been linearized by NheI/XhoI digestion (New England Biolabs). The primers used for cloning were as follows: FOXM1re1-F-Nhe1: 5′ – GCGGGCTAGC AACTGAGATTTGAAGGTAGAGGTGTG - 3′, FOXM1re1-R-Xho1: 5′ – GATACTCGAGCATTTTAGTACTTGCATGTGGTTAT- 3′, FOXM1 re2/3-F-Nhe1: 5′ – GGTCGCTAGCTGCCTGGAGTATTGCAACATCCAAC- 3′, FOXM1re2/3-R-Xho1: 5′ – GATACTCGAGGAGCGTTAAGGTCACGTGACGGAAC- 3′. All constructs were completely sequenced. For luciferase assays, a total of 1.2×10^6^ H1299 cells were seeded in 12-well dishes 24 h before transfection. In total, 100 ng of pGL3 reporter vector, 2 ng of pRL-CMV-*Renilla* luciferase vector (Promega) and 300 ng of HA-ΔNp63α expression vectors or empty pcDNA-HA vector (as a control) were cotransfected using the Effectene transfection reagent according to the manufacturer's instructions (Qiagen). The luciferase activities of cellular extracts were measured 24 h after transfection using a Dual Luciferase Reporter Assay System (Promega). The light emission was measured over 10 sec using a Lumat LB9507 luminometer (EG&GBerthold). The transfection efficiency was normalized to *Renilla* luciferase activity.

### Immunofluorescence

Briefly, paraffin-embedded sections of normal human skin samples were cut, then incubated for 30 min at 60°C, then washed with limonene 3 times and hydratated by immersing subsequently in 100%, 90%, 80%, 70%, and 50% ethanol solutions. Then samples were boiled for 10 min in 10 μM solution of sodium citrate (Sigma) and incubated in 0.1 M solution of sodium tetrahydroborate overnight at 4°C. Then samples were washed once with PBS and permeabilized with 0.30% Triton-X-100 in PBS for 30 min. Samples were blocked with 10% goat serum in PBS for 1 h and then exposed to primary antibodies. Samples were treated with anti-FOXM1 (Ab184637, Abcam, 1:20), anti-p63 (Ab735, Abcam, 1:50), anti-Keratin10 (PRB-159P, Covance; 1:1000), and anti-Keratin14 (PRB-155P, Covance; 1:1000) primary antibodies overnight at 4°C. Samples were washed 3 times with PBS and then treated with a 488- or 568-Alexa Fluor secondary antibodies (1/1000 dilution; Invitrogen) and DAPI for 1 hour. After three washes in 1X PBS, the slides were mounted using the Prolong Antifade kit (Invitrogen). Slides were analyzed with a confocal laser microscope (NIKON Eclipse Ti). Detection of the signal was performed using EZ C.1 software (Nikon).

### Senescence-associated β-galactosidase staining

Cells were grown in 60-mm culture dishes, washed with PBS, and fixed with 2% formaldehyde/0.2% glutaraldehyde/2 mM MgCl_2_ in PBS for 5 min. After another washing step with PBS, cells were incubated with β-galactosidase staining solution (2 mM MgCl_2_, 5 mM potassium ferricyanide, 5 mM potassium ferrocyanide, and 1 mg/mL 5-bromo-4-chloro-3-indolyl-β-D-galactoside [X-gal], pH 6.0) for 24 h at 37°C. The reaction was stopped by replacing the staining solution with 70% glycerol.

### Cell proliferation

The incorporation of EdU during DNA synthesis was evaluated using the Click-iT EdU flow cytometry assay kit according to the manufacturer's protocol (Molecular Probes). The cell cycle was analyzed using an Accuri C6 flow cytometer (BD Biosciences). Fifteen thousand events were evaluated using the Accuri C6 (BD) software.

### Measurement of ROS level and cell death

NHEKs were treated with H2O2 (Sigma) or doxorubicin (Sigma) at the indicated concentrations. Cells were collected 24 h after exposure, washed with PBS and then stained with 10 μM CM-H2DCFDA (Life Technologies) for 20 min at 37°C, followed by analysis using a BD FACSCalibur flow cytometer (BD Biosciences). For cell death measurements, cells were collected with culture medium, treated with 13 kU/ml RNAse (Sigma) for 15 min at 37°C and then with 50 μg/ml propidium iodide (Sigma) for 20 min, followed by flow cytometry analysis.

### Bioinformatic analysis

The analysis of the 5′ regulatory region of FOXM1 was performed using p63scan software. The p63scan algorithm can be downloaded from http://www.ncmls.eu/bioinfo/p63scan [59].

## SUPPLEMENTARY DATA FIGURES



## Abbreviations

FOXM1: forkhead box protein M1
ROS: reactive oxygen species
TF: transcription factor
SOD2: manganese superoxide dismutase 2
CAT: catalase
GPX2: glutathione peroxidase 2
PRDX: peroxiredoxin
Keratin 14: K14 (KRT14)
Keratin 10: K10 (KRT10) RE, response element
NHEK: normal human epidermal keratinocyte
Rb: retinoblastoma protein
