# Supplementary material for: FOXM1 regulates proliferation, senescence and oxidative stress in keratinocytes and cancer cells
Source: Aging (Albany NY). 2016 Jul 3;8(7):1384–96. doi: 10.18632/aging.100988 (PMC4993337; doi:10.18632/aging.100988)
Supplement: Supplementary file 1 [file aging-08-1384-s001.pdf]

wide profiling of p63 DNA-binding sites identifies an element that regulates gene expression during limb development in the 7q21 SHFM1 locus. PLoS Genet. 2010; 6:100106.

SUPPLEMENTARY DATA

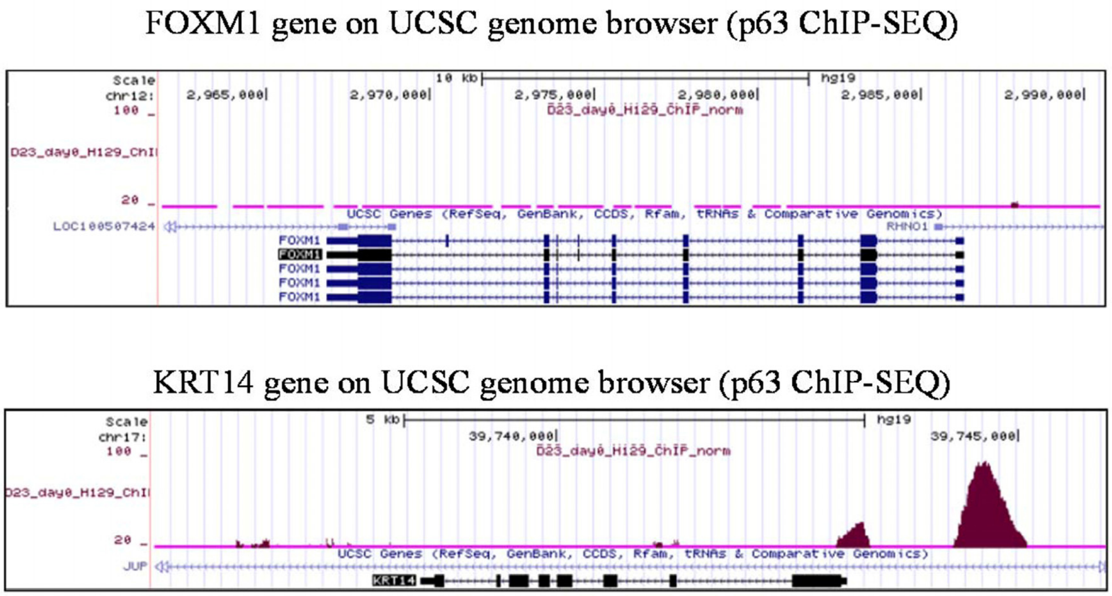

Figure S1. A screen-shot from UCSC genome browser (https://genome.ucsc.edu/) showing the peaks of p63-ChIP-seq performed in proliferating keratinocytes (GSE59827). *KRT14* gene was used as a positive control.

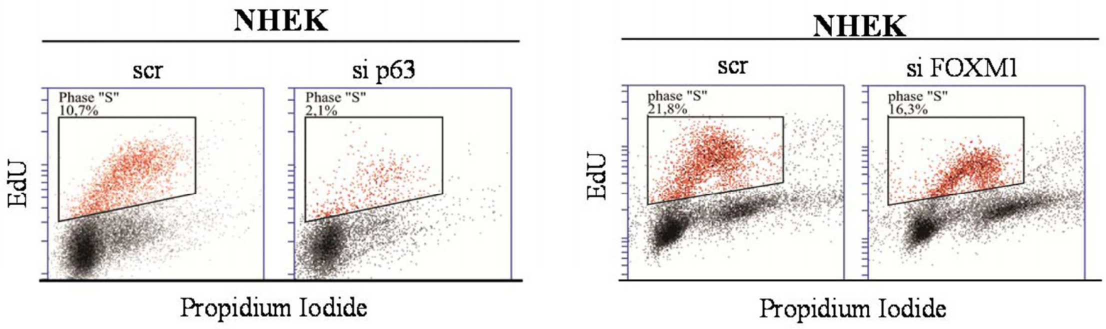

Figure S2. Dot-plots representing quantification of EdU-positive cells by FACS in NHEK silenced for sip63 or FOXM1.

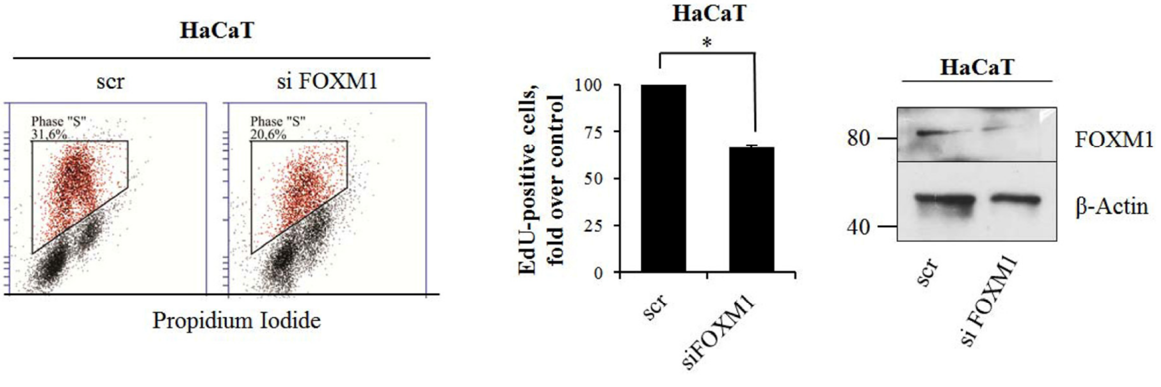

Figure S3. FOXM1 level correlates with proliferation status of keratinocytes. HaCaT cells were silenced for FOXM1 for 96h and then EdU-incorporation assay was performed. Percentage of EdU-positive cells was analysed by FACS. Western blots confirm the silencing. Values reported are the average ± SD of two independent experiments. *p-Value <0.01 by Student's t-test.
